# Evaluation of Silibinin Effects on the Viability of HepG2 (Human hepatocellular liver carcinoma) and HUVEC (Human Umbilical Vein Endothelial) Cell Lines

**Published:** 2018

**Authors:** Niki Vakili Zahir, Maryam Nakhjavani, Parastoo Hajian, Farshad H Shirazi, Hamidreza Mirzaei

**Affiliations:** a *Department of Toxicology and Pharmacology, school of Pharmacy, Shahid Beheshti University of Medical Science, Tehran, Iran. *; b *Cancer Research Center, Shohadae Tajrish Hospital, Department of Radiation Oncology, Shahid Beheshti University of Medical Sciences, Tehran, Iran. *; c *Department of Toxicology and Pharmacology, school of Pharmacy, Shahid Beheshti University of Medical Science, Tehran, Iran. *; d *SBMU Pharmaceutical Sciences Research Center, Tehran, Iran.*; e *Cancer research Center, Shohadae Tajrish Hospital, Department of Radiation Oncology, Shahid Beheshti University of Medical Sciences, Tehran, Iran.*

**Keywords:** Silibinin, Human hepatocellular carcinoma, HUVEC, HepG2

## Abstract

Human hepatocellular carcinoma is one of the most common recurrent malignancies since there is no effective therapy for it. Silibinin, a widely used drug and supplement for various liver disorders, demonstrated anti-cancer effects on human hepatocellular carcinoma, human prostate adenocarcinoma cells, human breast carcinoma cells, human ectocervical carcinoma cells, and human colon cancer cells. Considering the anti-hepatotoxic activity of silibinin and its strong preventive and anti-cancer efficacy against various epithelial cancers, we investigated the efficacy of silibinin against human HCC and HUVEC cell lines. Silibinin effects on the growth and mode of cell death of these two cell lines are presented in this paper. HepG2 and HUVEC cells were incubated with different doses of silibinin (12.5, 25, 50, 100, 150 and 200 μg/mL) at 24, 48, and 72 h. Cytotoxicity was assessed using MTT and Trypan blue assays. Mode of cell death induced by silibinin was investigated using LDH assay and acridine orange/PI double dye staining. The results showed that silibinin has dose-dependent inhibitory effect on the viability of HepG2 and HUVEC cells. However, Silibinin causes a more continuous dose-dependent cytotoxicity in HepG2 cells compared to the HUVEC cells in which some degrees of resistance is apparent at the beginning. The mode of cell death looks also different in these two cell lines with HepG2 cells being more in favor of apoptosis while necrosis is more evident for the HUVEC cells.

## Introduction

Cancer is one of the leading killers of human being in the world, which most likely happens as the result of different causes such as mutagenic and carcinogenic chemicals in the environment (1). Hepatocellular carcinoma (HCC) is one the most common cancers with more than 1 million fatalities occurring annually worldwide (2), and thus looking for newer therapeutics agents to treat this illness is still an ongoing concern for many researchers. 

Antioxidants have long been as effective agents in prevention and medication of many diseases including cancer. Therefore, many fruits and vegetables, which are sources of abundant antioxidants (3), are good candidate for pharmacological investigations. Certain foods have been shown to offer significant protection against various cancers, although their molecular mechanisms of protection are not yet completely known (3-7). Silibinin, a standardized milk thistle extract, is widely consumed as dietary supplements (8-10). It is one of the most interesting known antioxidants to date (11-13). Some of researches have tried to investigate on the cytotoxicity effects of silibinin on cancer cells. Several studies have shown that silibinin is a very strong compound, which enhances cellular antioxidant defense machinery of cells (15-18). This might prove to be a significant tool in the prevention of cancer growth and metastasis, and in reducing the risk of cancer. Researchers have also evaluated the anti-angiogenic efficacy of silibinin in human endothelial cells (19). Silibinin has been shown to inhibit human prostate tumor xenograft through the increase of apoptosis as well as the inhibition of tumor angiogenesis (20). In many animal studies, no apparent toxicity has been observed in the oral administration of silibinin (19). In addition, other studies have shown the hepatoprotective and nephroprotective effects of silibinin against various toxins (19-21). Many studies have proved the growth inhibition of many types of cancer cells derived from skin, prostate, breast, lung, cervix, colon, and blood (20, 22-28). Silibinin is already in clinic as hepatoprotective drug (21). 

Controversial results are observed from different publications on the cytotoxicity and/or modes of cell death induced by this agent in different cell lines (10, 18, 19, 22-33). Hepato-protectivity effects of this agent has also been mentioned in some articles (11, 15, 16, 21). To further investigate and clarify these controversial findings, we have therefore investigated on the cytotoxicity effects and mode of cell death or protection induced by this agent in two different human hepatic and endothelial cell lines whose results are presented in this paper. 

## Experimental

*Materials*

All the compounds used in this study were purchased form Sigma, USA, and otherwise mentioned in the text.

*Cell Culture and Silibinin Treatment*

Human hepatocarcinoma cell line (HepG2) and human umbilical vein endothelial cell line (HUVEC) were obtained from the National Cell Bank of Iran (NCBI). The cells were cultured in DMEM media containing 10% FBS, under a humid atmosphere (37 °C, 5% CO_2_, 95% air). For silibinin treatment, 1 mg/mL stock solution of silibinin was prepared in dimethyl sulfoxide (Merck, Germany). The stock was then mixed with appropriate amounts of media to expose the cells with doses of 12.5, 25, 50, 100, 150, and 200 μg/mL for 24, 48, and 72 h to any of above mentioned cell lines. Further to exposure, the following tests were performed on the cells.

*Determination of Cell Viability*

The effect of silibinin on HepG2 and HUVEC cell viability was determined using MTT assay. Briefly, 1×l0^4^cells/well was treated with 0-200 μg/mL doses of silibinin. After 24, 48, and 72 h of incubation, MTT (0.5 mg/mL PBS) was added to each well and incubated at 37 °C for 3 h. The formed formazan crystals were dissolved in dimethyl sulfoxide (150 μL /well) and the absorbance was read at 570 nm using a microplate scanning spectrophotometer (ELISA reader, OrganonTeknika, Netherlands). 

*Viable Cell Count*

Exponentially growing cells seeded in 12-well plates were trypsinized, washed twice with PBS and 50 µL of cell suspension (obtained from treated and non-treated HepG2 and HUVEC cells) was mixed with 50 µL of 0.4% trypan blue dye and left for 5 min at room temperature. Trypan blue exclusion was measured using a haemocytometer and the percentage of viable cells was determined.

*LDH Assay*

To determine the effect of silibinin on membrane permeability in HepG2 cell line, lactate dehydrogenase (LDH) assay was performed. 1×10^4 ^cells were grown in 96-well plates. 50 µL of culture media supernatant was transferred to a well of a 96-well plate and mixed with 50 µL of the substrate. Then, to determine LDH release, 50 of 6% triton X-100 was added to the original plate for the determination of total LDH. The absorbance was measured after the suggested reaction time at 490 nm. The percentage of LDH release was determined by dividing the LDH released into the media by the total LDH following cell lysis in the same well. The positive control cells used to show total LDH release were treated with 1% Triton X-100 in culture media. 

*Acridine Orange/Propidium Iodide Double-Staining Assay*

The possible apoptotic cell death mode induced by silibinin was evaluated by acridine orange-propidium iodide staining, followed by fluorescence microscopy analysis. 10 μL of the staining solution (10 μg/mL of acridine orange and 10 μg/mL of propidium iodide) was added to 10 μL of cell suspension (exposed to 100 µg/mL silibinin). Glass slides of the freshly stained cells were observed under a UV-fluorescence microscope to look at the incidence of cells stained by each dye.

*Statistical analysis*

The data were evaluated statistically using one-way ANOVA and Dunnet comparison test. Data are given as the means ± standard deviations (SD) of three to four determinations. Significance level was considered as *p *<0.05.

## Results

**Figure 1:**
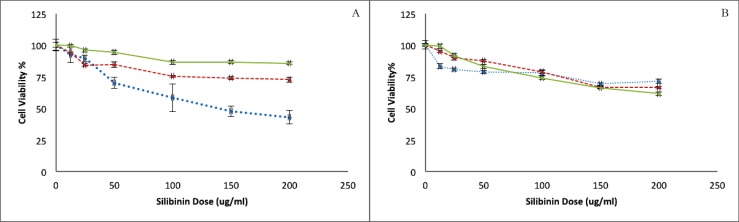
MTT assay on A) human hepatocarcinoma cell line and B) human umbilical vein endothelial cell line exposed to different concentrations of Silibinin for 24 (dotted), 48 (dash) and 72 h (line). Cell survivals are significantly different from each other in all concentrations with a *p* <0.05

Cytotoxicity of silibinin on HepG2 and HUVEC cells was assessed using a MTT assay (Figure 1A and 1B). As is shown in Figure 1, Silibinin could cause concentration-dependent cell death in both of malignant HepG2 and non-malignant HUVEC cell lines. However, the level of cytotoxicity in HUVEC cells is barely reaching 25% even after the exposure of 200 μg/mL of silibinin for 72 h. This effect is, however, not time dependent in either cell lines. HepG2 cells seem to be more sensitive to silibinin at shorter exposure times, and highest sensitivity to MTT assay was observed in this cell line at the highest exposed concentration during the shortest exposure time.

**Figure 2:**
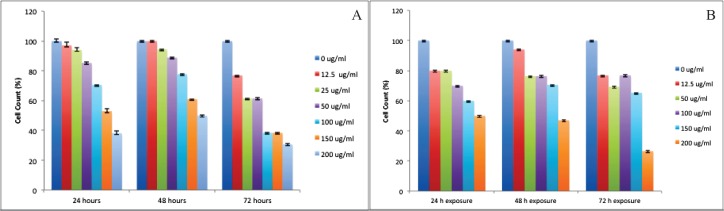
Trypan Blue die exclusion assay on A) human hepatocarcinoma cell line and B) human umbilical vein endothelial cell line exposed to different concentrations of Silibinin for 24, 48 and 72 h. Cell survivals are significantly different from each other in all concentrations with a *p*<0.05

**Figure 3:**
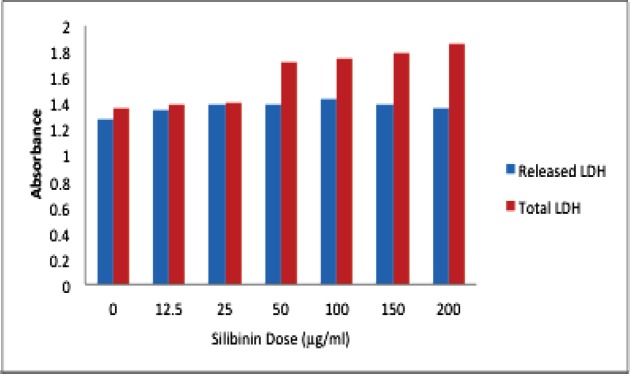
LDH release assay on human hepatocarcinoma cell lineexposed to different concentrations of Silibinin for 24. Cell survivals are significantly different from each other in all concentrations with a *p* <0.05

**Figure 4:**
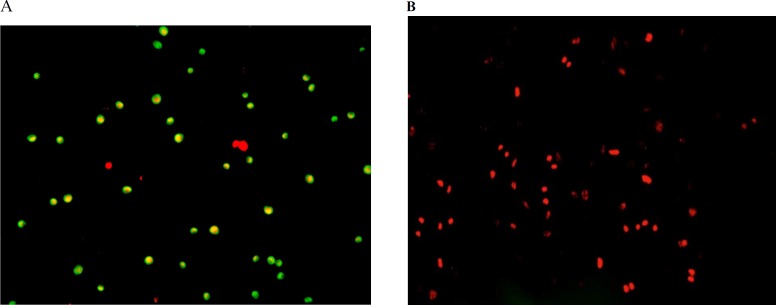
Fluorescence spectroscopy examination onhuman hepatocellular carcinoma (A) andhuman umbilical vein endothelial (B) cells after 24 h exposure to 100 µg/mL of Silibinin. Cells express more of apoptotic cell death (A; green dotted cells) at the beginning followed by secondary necrosis (B; red dotted and homogeny cells) afterthe exposure to Silibinin

The MTT assay on non malignant HUVEC showed a concentration dependent cytotoxicity whereas the significant differences were not observed between the results for different exposure times. In the comparison of two studied cell lines, the sensitivity of HepG2 is higher than HUVEC to silibinin. 

We also observed death-inducing effect of silibinin by trypan blue dye exclusion method (Figure.2A and Figure.2B). There was a significant difference between silibinin effect on growth inhibition of HepG2 and HUVEC cells at 24, 48, and 72 h (*P *<0.01).

Treatment of HepG2 cells with the different concentrations of silibinin resulted in a dose- and exposure time-dependent inhibition of cell growth as 60% (for 24 h exposure), 50% (for 48 h exposure), and 65% (for 72 h exposure). The comparison between 24 to 72 h exposure data showed that HepG2 cell viability was not decreased with increasing exposure time from 24 h to 48 h. Figure 2A demonstrated the HepG2 cell viability after exposure to silibinin for 24 to 72 h. Treatment of HUVEC cells with different concentrations of silibinin resulted in a dose- and time-dependent inhibition of cell growth amounting to 50% (for 24 h exposure), 53% (for 48 h exposure), and 70% (for 72 h exposure). The comparison between 24 to 72 h exposure data showed that HUVEC cell viability was also decreased with increasing exposure time (Figure 2B).

It appears that HepG2 and HUVEC cells reacted differently to silibinin exposure in the trypan blue dye exclusion assay. HUVEC cells appeared to be more sensitive to silibinin. Cells count results indicated that viability of HUVEC cells was significantly inhibited by silibinin at 12.5 µM concentration.

Silibinin exposure to HepG2 cells raised LDH leakage of these cells from 30% to 45% at different concentrations (Figure 3).

Furthermore, we evaluated the possible apoptotic or necrotic modes of cell death caused by silibinin in these two cell lines using acridine orange-propidium iodide staining and fluorescence microscopyanalysis. As is shown in Figure 4, while more of apoptotic cell death is apparent under the fluorescence microscope for HepG2 cells after exposure to silibinin, most of the HUVEC cells died by this agent have penetrated PI easily as the indication of necrotic death.

## Discussion

Many scientists are now interested in examining the use of herbal medicines as a health care method (13). Developments of biologically targeted agents that exploit differences between cancerous and normal cells with plant derived and less damage to normal cells are still the ultimate goal in the field of antineoplastic drug discovery (15). It is important to compare the cytotoxicity of a novel compound between cell lines and even with other commercial cytotoxic agents.

In this study, we showed that silibinin is cytotoxic against both of HepG2 and HUVEC cell lines in the studied concentrations. We also revealed that the inhibition of silibinin on HepG2 cell growth follow a dose-dependent linear pattern. Our study revealed no time dependency in MTT assay results for HepG2 cell line after exposure to silibinin.

Controversies are shown in published data for silibinin cytotoxicities in different cell lines. Yousefi *et al.* has shown that the inhibitory effect of silibinin on metabolic activity of metastatic human breast cancer cell line, SKBR3 (ErbB2-overexpressed and ER-negative breast carcinoma cell line) by MTT assay is concentrations and time intervals (24, 48 and 72 h) dependent (31). and Ge *et al. *has also shown that the cytotoxicity of Silibinin on human pancreatic cancer cell line AsPC-1, Panc-1 and BxPC-3, by MTT assay follow a concentration- and time-dependent manner (32). In the contrary, Li Jin study of silibinin on esophageal squamous cell carcinoma proliferation on two cell lines of KYSE 270 and T.Tn using MTT and colony forming assays failed to present a significant cytotoxic effect or pro apoptotic effect on these cell lines (33). 

In our study, while a significant cytotoxicity of sibilinin is shown on HepG2 cells, but HUVEC cells are only about 25% died by sibilinin even after exposure to the highest concentration of 200 μg/mL.

Necrotic cell death is almost the dominant pattern in HUVEC cell line. Since a variable of apoptosis and necrosis have been recognized in HepG2 cells, we have conducted LDH assay to confirm the necrotic variation mode of cell death in this cell line. We have also found increasing lactate dehydrogenase (LDH) release to the media pattern, after exposure to silibinin for 24, 48, 72 h in HepG2 cell line. 

Based on the results of the trypan blue dye exclusion assay, increasing silibinin exposure time had a detrimental effect on HepG2 and HUVEC cell viability. In our study, silibinin at IC_50_ doses for HepG2 and HUVEC cells also resulted in reduced growth of HepG2 and HUVEC cells with trypan blue assay test, confirming the previous reports at hepatocellular cell lines (31,32), These results promoted to further test silibinin for HCC chemoprevention. 

Furthermore, we evaluated the possible apoptotic cell death effect of silibinin by acridine orange-propidium iodide staining and fluorescence microscopy analysis, in which only acridine orange-stained cells were considered as apoptotic cells (Figure 4). Silibinin (100 µg/mL) treatment for 24 h caused more of necrotic cell death in HUVEC cells, but apoptotic death in the HepG2 cells.

In general, we have found necrosis as the dominant mode of cell death caused by silibinin. The delay LDH release after exposure of HepG2 cells to silibinin might be correlated to the secondary necrosis of these cells further to the late stage of apoptosis. A primary start in apoptotic cell death is evident in the double-dye staining of these cells which led to secondary necrosis by the time. HUVEC cells do not reach necrosis through an initial apoptotic pattern since these cells are more resistant and a higher concentration of silibinin for these cells causes necrotic cell death. 

As a conclusion, our results suggest a dual mechanism and mode of cell death in two different cell lines for silibinin; apoptotic and dose-dependent death for more sensitive hepatic cells, while necrotic at high concentrations death for endothelial cells. Further investigations are recommended to reveal the biological effects of this agent on different cell lines and tissues.
